# Characterization Method of Damage Information Based on Heterogeneous Network

**DOI:** 10.3390/s23136035

**Published:** 2023-06-29

**Authors:** Tong Huang, Qinhe Gao, Zhihao Liu, Dong Wang, Dong Ma, Lei Gao

**Affiliations:** National Key Discipline Laboratory of Armament Launch Theory & Technology, Rocket Force University of Engineering, Xi’an 710025, China; tongh201@126.com (T.H.); zhliu201@163.com (Z.L.); dwang201@163.com (D.W.); dma201@163.com (D.M.); lgao201@163.com (L.G.)

**Keywords:** damage process, damage flow, heterogeneous network, network flow theory

## Abstract

Damage is the main form of conflict, and the characterization of damage information is an important component of conflict evaluation. In the existing research, damage mainly refers to the damage effect of a damage load on the target structure. However, in the actual conflict environment, damage is a complex process that includes the entire process from the initial introduction of the damage load to the target function. Therefore, in this paper, the transfer logic of the damage process is analyzed, and the damage process is sequentially divided into being discovered, being attacked, being hit, and being destroyed in succession. Specifically, first considering the multiple types of each process, the transmission of damage is likened to the flow of damage, a network model to characterize damage information based on heterogeneous network meta-path and network flow theory (HF-MCDI) is established. Then, the characteristics of damage information are analyzed based on the capacity of the damage network, the correlation of the damage path, and the importance of the damage node. In addition, HF-MCDI can not only represent the complete damage information and the transmission characteristics of the damage load but also the structural characteristics of the target. Finally, the feasibility and effectiveness of the established HF-MCDI method are fully demonstrated by the example analysis of the launch platform.

## 1. Introduction

In the existing research, damage mainly refers to the damage effect of a damage load on the target structure. For example, the damage response of structures under damage loads, such as shock waves or fragments [1], damage effect of energetic fragments on a target plate [2], and other structural damage characteristics in the conflict environment [3]. However, in the actual conflict environment, damage is a complicated transmission process that includes the whole process from the launch of the damage load to the target function. This process includes: (1) After reconnaissance finds the target, determining whether or not to attack; (2) After determining the target value, initiating the damage charge to the target; (3) After the target evades protection, the damage charge begins to impact the target structure; (4) Until the core structure is destroyed, determining the target is destroyed. Therefore, how to systematically characterize this logical process becomes the key to operational assessment.

At present, the characterization of damage information mainly includes test methods and model methods. Among them, the reliability of the results of the experimental method is high [4], but the research cost is also relatively high, and for some complex systems, it is even difficult to conduct experimental studies. Model methods are divided into two categories according to the underlying theory: evaluation methods based on mathematical statistics [5,6] and knowledge inference [7,8], among which the evaluation methods based on mathematical statistics have a simple model-building process and easy calculation and are widely used for the characterization of destructive information in areas such as vulnerability and conflict effectiveness [9]. The evaluation method based on knowledge inference is relatively higher than the evaluation method based on mathematical statistics in terms of evaluation accuracy and credibility [10], but it relies on prior knowledge and subjective judgment of experience, therefore, the calculation process is more complicated. In summary, the current method of representing damage information is influenced by subjective empowerment and probability statistics, and suffers from a lack of objectivity and physical interpretation, and has a poor correlation between the representation process and reality.

Network analysis is one of the research hotspots in the field of information representation. Most of the current information network analysis work is on homogeneous information networks, i.e., the same type of nodes and network link relationships [11]. Homogeneous information networks mainly extract part of the information of the interactive systems without distinguishing the differences between the interactive systems, which easily leads to the loss of semantic information extraction. Therefore, researchers have proposed heterogeneous information networks with multi-type nodes [12]. In addition, meta-path is an important concept defined in heterogeneous information networks to extract semantic information from heterogeneous networks [13,14]. Compared to homogeneous information networks, heterogeneous information networks contain more comprehensive structural information and rich semantic information.

**Figure 1:**
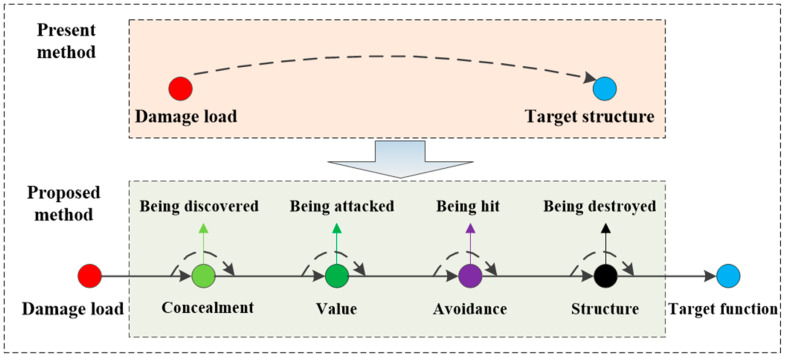
The comparison of the characterization method between proposed and existing.

Based on the above description, this paper first proposes a network model to characterize damage information based on heterogeneous network meta-path and network flow theory. This network model is divided into an efficiency attribute sub-network, an avoidance attribute sub-network, a structure attribute sub-network, and a function attribute sub-network according to the transmission logic of the damage process. Each sub-network is connected based on the network flow theory, and the damage process is represented by the damage network flow. The comparison between the proposed damage information characterization method and the existing damage information characterization method is illustrated in Figure 1. Then, the characteristics of the damage information are analyzed in terms of damage network capacity, damage path correlation, and damage node significance. HF-MCDI can represent not only the complete damage information and the transmission characteristics of the damage load, but also the structural characteristics of the target. The innovations and contributions of this research can be summarized as follows:

1. The proposed network model can represent the damage process more completely and is more a in line with the actual conflict environment;
2. The proposed network model is no longer attacker-centered, but defender-centered, which can analyze the structure–function characteristics of the defender in more detail, and the probability representation is no longer required.
3. The established damage capacity model, damage path model, and damage node model can evaluate the damage process more objectively.

The remainder of this article begins with network model building in Section 2. In Section 3, the analysis method of network model is constructed. The example analysis is carried out in Section 4. Section 5 provides general conclusions.

## 2. Network Model

### 2.1. Conceptions

**Definition** **1.** *Heterogeneous network [15]. In the digraph G=(V,E), V represents a set of nodes and E represents a set of edges. There is a node-type mapping function Ψ: V→ℜ, and an edge-type mapping function Φ: E→ℵ, where ℜ is node-type, ℵ is edge-type, and if there is |ℜ|+|ℵ|>2, this type of network is defined as a heterogeneous network, and S={ℜ,ℵ} is defined as network mode.*

**Definition** **2.** *Meta-path [15]. A meta-path is a path defined on a network mode S={ℜ,ℵ} as:*

(1) $$V_{1}\overset{R_{1}}{\to }V_{2}\overset{R_{2}}{\to }\ldots \overset{R_{l}}{\to }V_{l+1}$$

*This path describes a composite relationship between specific nodes $V_{l}$:*

(2) $$R=R_{1}\circ R_{2}\circ \ldots \circ R_{l}$$

*where ∘ represents the composition operator on the composite relation $R_{l}$.*

**Definition** **3.** *Network capacity [16]. c(e) is a non-negative function defined on the set E of connected edges, and if there are connected edges $e=v_{i}v_{j}$, then defined c(e) is the capacity on the connected edges e. Similarly, c(v) is defined as the capacity on the node v.*

**Definition** **4.** *Network flow [16]. f(e) is a function defined on a connected edge set E; if there is an edge $e=v_{i}v_{j}$, $f_{ij}=f(e)$ is defined as a flow on a connected edge e.*

Based on the above theoretical conceptions and the transfer logic of the damage process, the conceptions of the network model are defined as follows.

**Definition** **5.** *Damage node. This refers to the conditions that affect the load transfer between the failure load and the target function. The tolerance of each condition is defined as the damage capacity of the damaged node.*

**Definition** **6.** *Damage path. This refers to the connection edge of the damaged node through which the damage load is transferred to the target function. The load that can pass through each edge is defined as the damage flow.*

**Definition** **7.** *Damage information. This refers to all the contents contained in the directed heterogeneous network formed by the combination of damage load, damage node, and damage path.*

**Figure 2:**
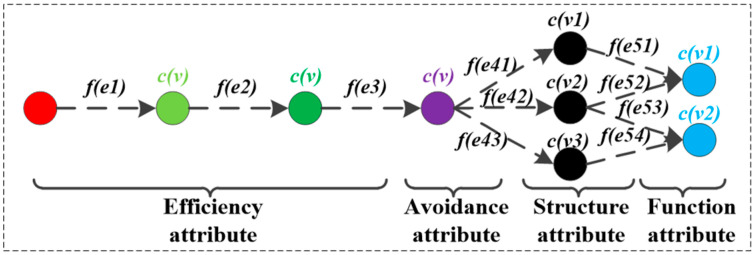
The framework of HF-MCDI.

Based on the above definition, the framework of HF-MCDI is shown in Figure 2. According to the attributes of damaged nodes, HF-MCDI is divided into four sub-networks. The efficiency attribute sub-network is composed of virtual nodes and logical associations that affect load transfer. The avoidance attribute sub-network consists of protection nodes and logical associations that affect load transfer. The structural attribute sub-network consists of structural nodes and installation associations that affect load transfer. The functional attribute sub-network consists of functional nodes that represent the results of load transfer. Additionally, the four sub-networks are elaborated in the following sections.

### 2.2. Efficiency Attribute Sub-Network

The efficiency attribute sub-network is defined as $G_{e}$. It can be seen from Figure 2 that the transmission of damage load in $G_{e}$ is tandem in nature and does not weaken. Therefore, $G_{e}$ has the following two characteristics:

1. There is only one path for the damage load to be transmitted in $G_{e}$, and the damage flow on the path was $f_{ij}=D$, where D is the damage load.
2. The damage capacity $c(v_{i})$ of damaged nodes is determined by node performance, and $c(v_{i})$ obeys 0−D step function distribution.

**Figure 3:**
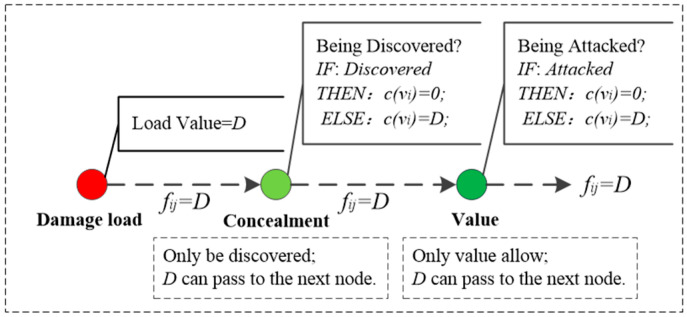
The framework of $G_{e}$.

In addition, the framework of $G_{e}$ is shown in Figure 3. For $G_{e}$ there is:

(3) $$\{\begin{array}{l} f_{eij}=D\\ c_{e}(v_{i})=\left\{ \begin{array}{l} 0,p<p_{0}\\ D,p\geq p_{0} \end{array}\right. \end{array}$$

where $f_{eij}$ is damage flow of $G_{e}$, $c_{e}(v_{i})$ is damage capacity of $G_{e}$, p is the conditional degree value of the damaged node, and $p_{0}$ is the critical point of the conditional degree value of the damaged node.

### 2.3. Avoidance Attribute Sub-Network

**Figure 4:**
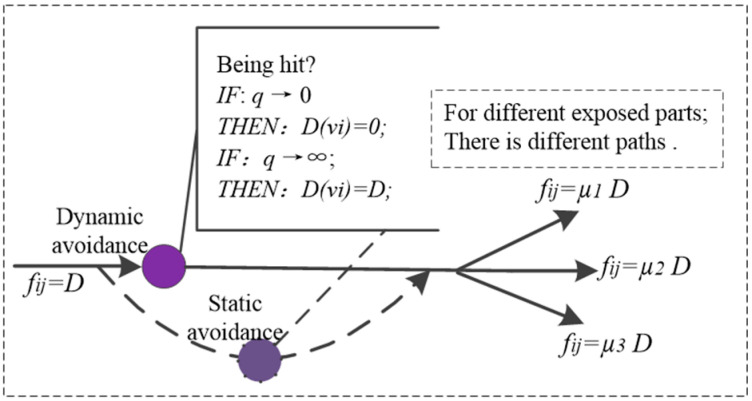
The framework of $G_{a}$.

The efficiency attribute sub-network is defined as $G_{a}$. The damaged node in $G_{a}$ is determined by the subjective judgment of the damage load avoidance amount. After the judgment, the damaged path will be divided into multiple paths according to the target structure. Therefore, $G_{a}$ has the following two characteristics:

1. The damage load avoidance amount $D(v_{i})$ is related to the target avoidance degree q, and obeys the logistic function distribution, which can be described as [17]:
  (4) $$\frac{dD(v_{i})}{dq}=QD(v_{i})[1-\frac{D(v_{i})}{D}]$$
  (5) $$D(v_{i})=\frac{1}{1+e^{-Q(q-q_{0})}}D$$
  where Q is the coefficient of the avoidance degree and $q_{0}$ is the midpoint of the avoidance degree.
2. The damage flow on the damage path is related to the exposed area of the target structure. If the number of exposed parts of the target is k, and $S_{k}$ is the exposed area of part k, then the path coefficient is $\mu_{k}=S_{k}/\sum_{k=1}^{k}S_{k}$.
  In addition, the framework of $G_{a}$ is shown in Figure 4. For $G_{a}$ there is:
  (6) $$\{\begin{array}{l} f_{aij}=\mu_{k}D\\ c_{a}(v_{i})=D(v_{i}) \end{array}$$
  where $f_{aij}$ is damage flow of $G_{a}$ and $c_{a}(v_{i})$ is damage capacity of $G_{a}$.

### 2.4. Structural Attribute Sub-Network

The structural attribute sub-network is defined as $G_{s}$. The modular arrangement of the structure makes $G_{s}$ hierarchical and clustered, and in $G_{s}$, the transmission of damage loads is a failure-resistant process of each node, and $G_{s}$ has the following characteristics.

1. The damage flow $f_{ij}$ along the damage path is the blocking effect of the structure on the damage load, and the smaller the blocking effect, the greater the damage flow.
2. Damage capacity $c(v_{i})$ is the extreme limit of deformation of a structure under a damage load. When the damage load transferred to the node is greater than the damage capacity of the node, the damage load overflows, and the remaining damage load continues to be transferred to the next node.

Therefore, assume that the structure is composed of a number of structural element units, the energy method is used to calculate the structural element deformation as [18]:

(7) $$\{\begin{array}{l} U_{1}=\frac{1}{2}∭(\sigma_{xx}\varepsilon_{xx}+\sigma_{yy}\varepsilon_{yy}+\sigma_{xy}\varepsilon_{xy})dxdydz\\ U_{2}=\frac{1}{2}\iint (N_{x}\varepsilon_{x}+N_{y}\varepsilon_{y}+N_{xy}\varepsilon_{xy})dxdy \end{array}$$

where $U_{1}$ and $U_{2}$ are bending strain energy and midplane strain energy. For rigid plastic materials: $\sigma_{xx}=\sigma_{yy}=\sqrt{3}\sigma_{xy}=\sigma_{s}$, $\sigma_{s}$ is the yield limits of materials. The mask force $N_{x}$, $N_{y}$ and $N_{z}$ are equal to $h\sigma_{x}$, $h\sigma_{y}$ and $h\sigma_{xy}$. h is the material thickness.

The strain energy of structural element is calculated as:

(8) $$\begin{array}{l} U=U_{1}+U_{2}\\ \;\;\;=f_{1}(a,b,h)\sigma_{s}W_{0}+f_{2}(a,b,h)\sigma_{s}W_{0}{}^{2}\\ \;\;\;=f_{1}(a,b,h)\sigma_{s}•\left[1,\frac{f_{2}(a,b,h)}{f_{1}(a,b,h)}\right]•\left[\begin{array}{l} W_{0}\\ W_{0}{}^{2} \end{array}\right] \end{array}$$

where $f_{i}(a,b,h)$ is the size function, and $W_{0}$ is the deformation deflection of the structural center.

When the damage load transferred to the structural element unit is $D_{0}(v_{i})$, let $\Psi =1/f_{1}(a,b,h)\sigma_{s}$ represent the blocking effect of the structural element unit on the damage load, and $Ζ={\left[W_{0},W_{0}{}^{2}\right]}^{T}$ is the response state of the structural element.

Taking the structural unit as the reference, the displacement at the boundary is 0 and the angle is 0. In this paper, the bit triangular function is utilized to simulate the equations of flexural lines as:

(9) $$W=\frac{W_{0}}{4}(1+\cos\frac{\pi x}{a})(1+\cos\frac{\pi y}{b})$$

where a is the size of the material in direction x, and b is the size of the material in direction y.

According to the maximum deformation $W_{0\lim}$ and maximum deformation volume $V_{\lim}$ of the material and based on the following formula, the maximum strain energy of structural element $U_{\lim}$ can be calculated.

(10) $$\{\begin{array}{l} W_{0}\leq W_{0\lim}\\ \iint \frac{W_{0}}{4}(1+\cos\frac{\pi x}{a})(1+\cos\frac{\pi y}{b})dxdy\leq V_{\lim} \end{array}$$

**Figure 5:**
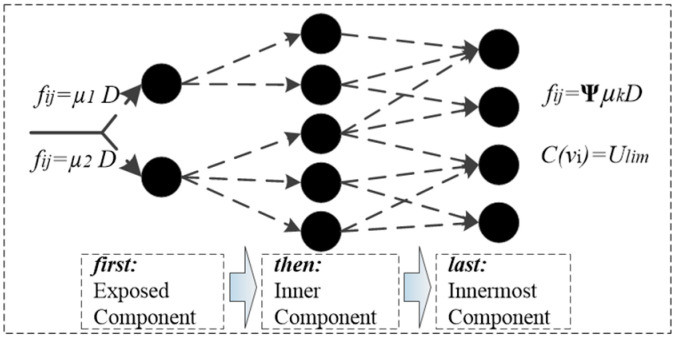
The framework of $G_{s}$.

In addition, the framework of $G_{s}$ is shown in Figure 5. For $G_{s}$ there is:

(11) $$\{\begin{array}{l} f_{sij}=\Psi \mu_{k}D\\ c_{s}(v_{i})=U_{lim} \end{array}$$

where $f_{sij}$ is damage flow of $G_{s}$, $c_{s}(v_{i})$ is damage capacity of $G_{s}$.

### 2.5. Functional Attribute Sub-Network

The structural attribute sub-network is defined as $G_{f}$. The functional attribute sub-network consists of functional nodes that display the results of load transfer. Since function is the display of structure, $G_{f}$ is the display of the running state of $G_{s}$. Therefore, for $G_{f}$ there is:

(12) $$\{\begin{array}{l} f_{fij}=D\\ c_{f}(v_{i})=0 \end{array}$$

In summary, a heterogeneous network model can be obtained to fully characterize the damage information, and a meta-path can also be obtained to characterize the transfer process of a damage load. The path $\rho =(V_{e},V_{a},V_{s},V_{f})$ is defined as the meta-path of the damaged information heterogeneous network, where $V_{e},V_{a},V_{s},V_{f}$ is the node set of $G_{e},G_{a},G_{s},G_{f}$. This meta-path is represented as:

(13) $$\underset{\left(c_{e}\right)}{V_{e}}\overset{\left(0,D\right)}{\to }\underset{\left(c_{a}\right)}{V_{a}}\overset{\mu_{k}D}{\to }\underset{\left(c_{s}\right)}{V_{s}}\overset{\Psi }{\to }\underset{\left(c_{f}\right)}{V_{f}}$$

## 3. Characteristics

### 3.1. Damage Capacity

**Figure 6:**
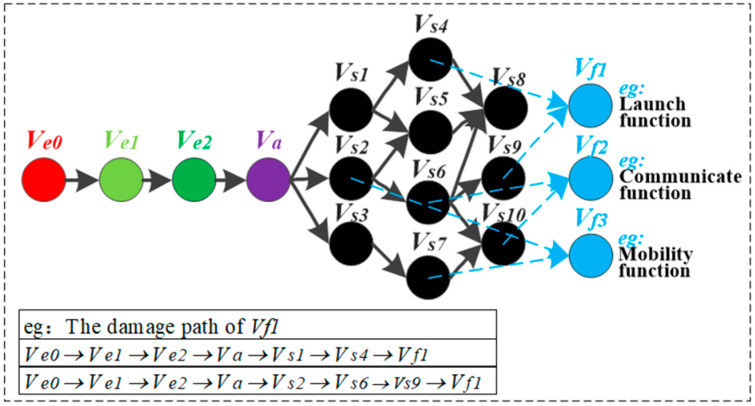
The damage path.

The damage path is shown in Figure 6; the starting point of the damage path of HF-MCDI is the damage load in $G_{e}$, which is defined as $V_{e0}$. The ending point is any functional node in $G_{f}$, which is defined as $V_{f\lambda }$. λ is the number of functional nodes. Then, there is:

(14) $$\forall V_{f\lambda },\exists \rho_{\lambda l}(V_{e0}\to V_{f\lambda }).$$

where $\rho_{\lambda l}$ is the damage path of Article l.

The damage capacity of each damage path is the sum of the damage capacities of nodes in different subnetworks on this damage path. That is, the damage capacity of $\rho_{\lambda l}$ is $c_{\lambda l}=[c_{e},c_{a},c_{s},c_{f}]$, where $c_{e},c_{a},c_{s},c_{f}$ are the damage capacity vectors of nodes of the damage paths in different subnetworks. When the damage capacity coefficient is defined as the ratio of damage capacity to damage load, $c_{l}=c(v_{i})/D$, the damage capacity coefficient matrix of the damage path can be established as $C=[c_{1};c_{2};\ldots ;c_{l}]$.

Then, the residual damage load coefficient transfer to the function on the damage path is:

(15) ΔD=1−sum(C,2)

where sum(C,2) is the sum of the row vectors of C.

Obviously, when ΔD=0 appears, it means that the damage capacity of the damage path is filled by the damage load, and the function of the target is in a critical state of damage. When ΔD<0 appears, the damage load exceeds the damage capacity, and the target function is damaged. When ΔD>0 appears, the damage load is blocked by the damage capacity, and the function of the target is not damaged.

### 3.2. Correlation of Damage Path

The correlation of the damage path can be used to measure the similarity of the damage load to the function; the higher the correlation of a damage path, the more dangerous this damage path is. In network flow theory, the damage flow on the damage path $f_{ij}$ is the weight of the connecting edges between nodes, and the weight matrix can be defined as $f_{l}{}^{*}=[f_{e},f_{a},f_{s},f_{f}]$. When the weight matrix is mapped to the same space, according to the subnetwork, it can be obtained by:

(16) $$f_{\tau l}=\frac{f_{\tau l}{}^{*}}{\sum_{\tau \in (e,a,s,f)}f_{\tau l}{}^{*}}$$

where $f_{\tau l}$ is the weight value after $f_{\tau l}{}^{*}$ mapping, and $f_{\tau l}{}^{*}\in f_{l}{}^{*}$.

Assume that $s_{i}(i=0,1,\ldots ,S)$ is the node number on the damage path, and $N_{si}{}^{\rho }$ is the neighbor node of node $s_{i}$ on the damage path $\rho_{\lambda l}$. The WsRel method is a typical path measurement method [19], which is suitable for obtaining accurate information between the source node and the target node. Based on the WsRel method, the correlation of damage paths is defined as:

(17) $$\begin{array}{l} WsRel(s_{0},s_{S}\;|\;f_{1l}f_{2l}\ldots f_{\tau l})=\\ \begin{array}{ccc} & & \frac{1}{\left|N_{si}{}^{\rho }\right|}\sum_{s_{1}\in N_{si}{}^{\rho }}f_{1l}•WsRel(s_{1},s_{S}\;|\;f_{2l}f_{3l}\ldots f_{\tau l}) \end{array} \end{array}$$

Since the HF-MCDI has many damage paths, the correlation of the HF-MCDI is:

(18) $$R=\sum_{l=1}^{l}\theta_{l}•WsRel(s_{0},s_{S}\;|\;\rho_{\lambda l})$$

where $\theta_{l}$ is the weight of the damage path in Article l, and $\theta_{l}$ is related to the connection strength ε. According to the Shi algorithm, ε in Article l is calculated as:

(19) $$\varepsilon_{l}=\prod_{i=0}^{S}\frac{1}{\sqrt{out\deg(s_{i})•in\deg(s_{i+1})}}$$

where outdeg(⊙) is out degree and indeg(⊙) is in degree. Additionally, the connection strength of the damaged path can be defined by the Softmax function as:

(20) $$\theta_{l}=soft\max(\varepsilon_{l})=\frac{\varepsilon_{l}}{\sum_{l=1}^{l}\varepsilon_{l}}$$

### 3.3. Importance of Damaged Nodes

The importance of the damaged node lies in its influence on the network model. Considering the heterogeneity of HF-MCDI, this paper selects the node shrinkage method to calculate the importance of the damaged nodes [20] and improves the node shrinkage method according to the correlation attributes of HF-MCDI. Considering the heterogeneity and rapidity of convergence of HF-MCDI, this paper selects the node shrinkage method to calculate the importance of the damaged nodes, improves the node shrinkage method according to the correlation attributes of HF-MCDI, and makes HF-MCDI more comprehensive.

The cohesion degree of HF-MCDI is defined as ∂[G], and ∂[G]=1/n•L, where n is the number of nodes in the network, L is the average path length, $L=\sum_{i\neq j\in V}d_{ij}/n(n-1)$, and $d_{ij}$ is the distance between node i and node j. The cohesion degree of HF-MCDI can be updated as:

(21) $$\partial [G]=\{\begin{array}{c} n-1/\sum_{i\neq j\in V}d_{ij}\begin{array}{cc} & n\geq 2 \end{array}\\ 1\begin{array}{ccc} & & \;n=1 \end{array} \end{array}$$

In addition, the importance of the damaged node is defined as:

(22) $$IMC^{*}(V_{i})=1-\frac{\partial [G]}{\partial [G*V_{i}]}$$

where $\left[G*V_{i}\right]$ is the network after node $V_{i}$ was shrunk.

Based on the above formula, there is:

(23) $$\begin{array}{c} IMC^{*}(V_{i})=1-\frac{1}{n•L[G]}/\frac{1}{\left(n-k_{i})•L[G*V_{i}\right]}\\ =\frac{n•L[G]-(n-k_{i})•L[G*V_{i}]}{n•L[G]} \end{array}$$

where $k_{i}$ is the number of neighbor nodes of $V_{i}$.

Due to the connectivity between neighboring nodes in HF-MCDI, it is not sufficient to consider only the number of neighboring nodes; the importance of neighboring nodes should also be considered. Therefore, the improved importance of damaged nodes is defined as:

(24) $$IMC(V_{i})=\eta IMC^{*}(V_{i})+\sum_{k\in N_{e}}\mu_{k}IMC^{*}(V_{N_{e}k})$$

where $V_{N_{e}k}$ is the neighboring node of $V_{i}$, η and $\mu_{k}$ are the proportional relationship of importance, $\eta +\sum_{k\in N_{e}}\mu_{k}=1$, which can be allocated based on damage capacity according to the following formula:

(25) $$\{\begin{array}{l} \eta =c(V_{i})/\left[c(V_{i})+\sum_{k\in N_{e}}c(V_{N_{e}k})\right]\\ \mu_{k}=c(V_{N_{e}k}{}_{i})/\left[c(V_{i})+\sum_{k\in N_{e}}c(V_{N_{e}k})\right] \end{array}$$

## 4. Analysis

### 4.1. Illustrative Example

**Figure 7:**
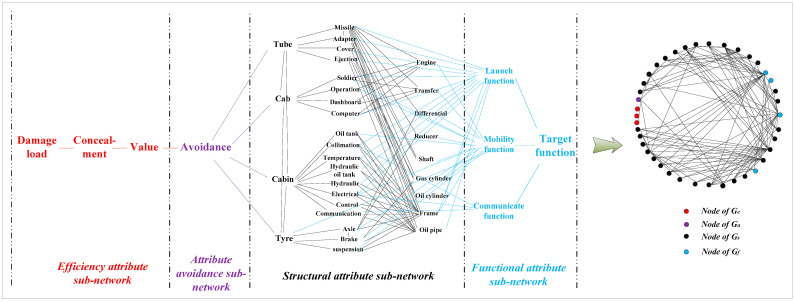
HF-MCDI of the special equipment platform.

In order to demonstrate the effectiveness of the proposed method in this paper, a special equipment platform was taken as the research object. The special equipment platform is a complex body combining structure and function. According to the establishment method of HF-MCDI in Section 2, a special equipment platform HF-MCDI with 4 node types, 40 network nodes, and 121 directed edges was established, as shown in Figure 7.

### 4.2. Basic Features of HF-MCDI

**Figure 8:**
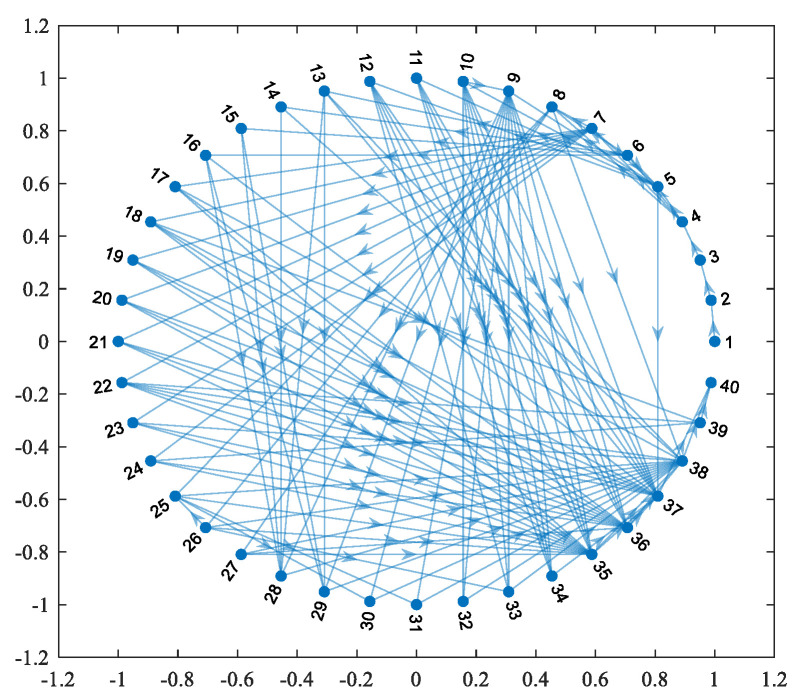
The visual description of HF-MCDI of the special equipment platform in MATLAB.

In this paper, MATLAB is used to calculate the model shown in Figure 7, where the nodes are numbered from 1 to 40, from left to right, and from top to bottom, and the visual description in MATLAB is shown in Figure 8. The basic characteristics of HF-MCDI are then analyzed in terms of degree distribution, clustering coefficients, and node centrality according to complex network theory.

#### 4.2.1. Degree Distribution

**Figure 9:**
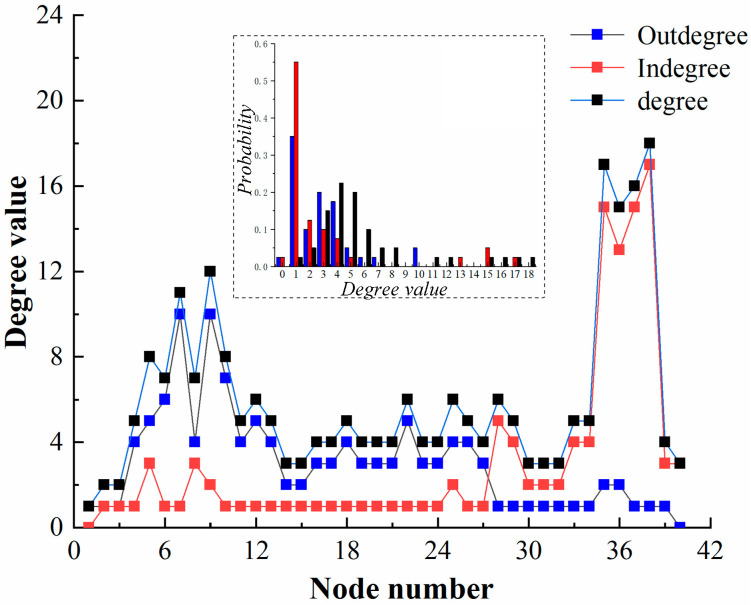
The degree distribution of HF-MCDI of the special equipment platform.

The degree distribution of HF-MCDI of the special equipment platform is shown in Figure 9.

Figure 9 shows that:

(1) The node degree of the network is mainly distributed in two parts: k>5 and k≤5. The proportion of k≤5 is 65%. Among them, k=4 has the largest proportion, which is 22.5%, and the nodes of k=5 and k=3 are 20% and 15%, respectively. The results show that there are 3–5 connecting edges in most nodes of this network, which accords with the characteristics of four-sided adjacency of structural components in space. The proportion of k>5 is 35%, indicating that there are some nodes with high passing frequencies in the network, which are the key to damage flow, and they are mainly distributed in the front of $G_{s}$ and $G_{f}$.
(2) The maximum indegree of the nodes in this network is in $G_{f}$, $\max(k_{in})=16$. The maximum outdegree of the nodes in this network is in $G_{s}$, $\max(k_{out})=10$. $k_{in}=5$ has the largest ratio, which is 55%, while $k_{out}<5$ has 85%. This shows that the transmission of damage information in this launch platform is mainly stratified and dispersed, which is determined by the integration and modularity of the launch platform.

#### 4.2.2. Clustering Coefficient

**Figure 10:**
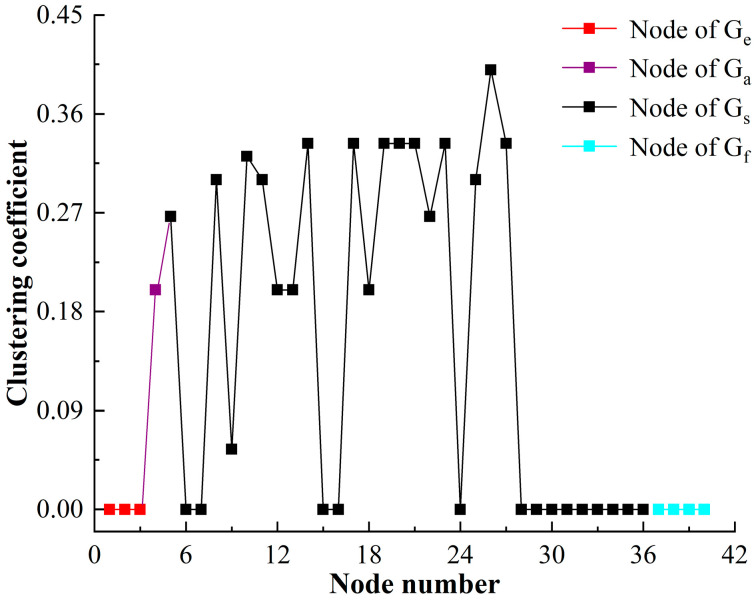
The clustering coefficient of HF-MCDI of the special equipment platform.

The clustering coefficient of HF-MCDI of the special equipment platform is shown in Figure 10.

Figure 10 shows that:

(1) The clustering coefficient of the network is 0 at nodes 1, 2, 3, and 28 to 40. The clustering coefficients of other nodes, especially those in $G_{s}$, all fluctuate around 0.25. The results show that there is a local aggregation phenomenon in the structure attribute subnetwork of the launching platform, and it is relatively uniform. When a node is damaged, it is easy to spread to the neighboring nodes, which is determined by the structural characteristics of the launching platform.
(2) The clustering coefficient is 0 in $G_{e}$. The result shows that the nodes in $G_{e}$ are not tightly connected because the concealment of the target and the value of the target don’t affect each other but are only logically connected, and this result is in line with reality.
(3) The clustering coefficient is 0 in $G_{f}$. Because the nodes in $G_{f}$ are connected to the nodes in $G_{e}$, and the nodes in $G_{f}$ are not related to each other, the aggregation of function is reflected in the aggregation of structure.

#### 4.2.3. Node Centrality

**Figure 11:**
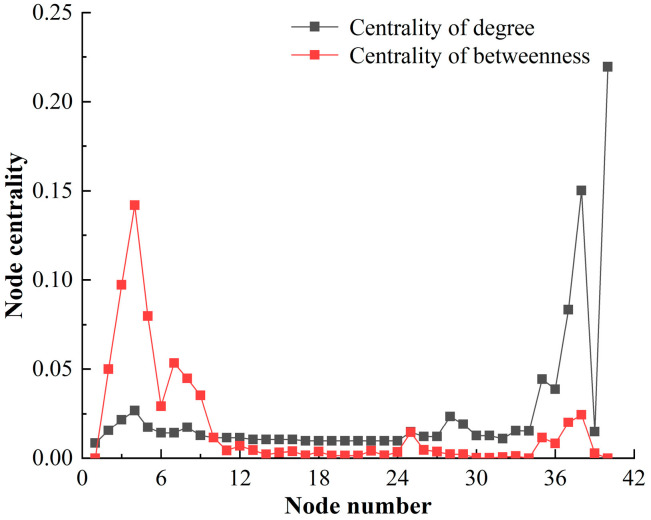
The node centrality of HF-MCDI of the special equipment platform.

The node centrality of HF-MCDI of the special equipment platform is shown in Figure 11.

Figure 11 shows that:

(1) From the centrality of degree: The node with the highest centrality of degree of the launch platform is the total function (0.2196), followed by the mobility function (0.1502) and the launch function (0.0834). This indicates that the node of $G_{f}$ has greater influence because the centrality of degree describes the centrality of nodes in the network, indicating that the launch platform operates according to the function. Therefore, the center of the network is concentrated in $G_{f}$.
(2) From the centrality of betweenness: The centrality of betweenness is distributed in three levels. ① There are 5 nodes with a centrality value greater than 0.05, including avoidance (0.1420), value (0.0973), tube (0.0798), concealment (0.0534), and cabin (0.0506); ② There are 3 nodes with a centrality value greater than 0.02 and less than 0.05, which are the exposed nodes of $G_{s}$; ③ The centrality value of the remaining 80% of the nodes is less than 0.02. This suggests that the avoidance node has the greatest impact due to the fact that mesoscopic centrality describes the role of damage paths, and avoidance is a critical node in the damage path of the special equipment platform.

### 4.3. Damage Capacity for HF-MCDI

**Figure 12:**
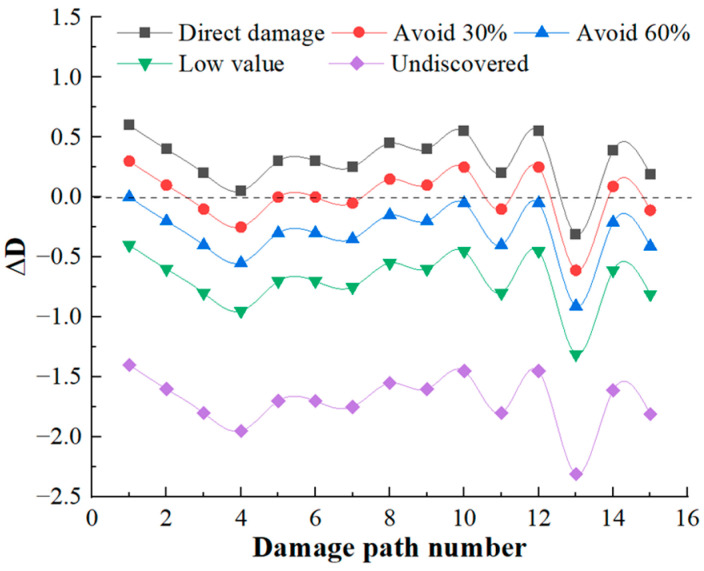
Residual damage load coefficient of launch function.

**Figure 13:**
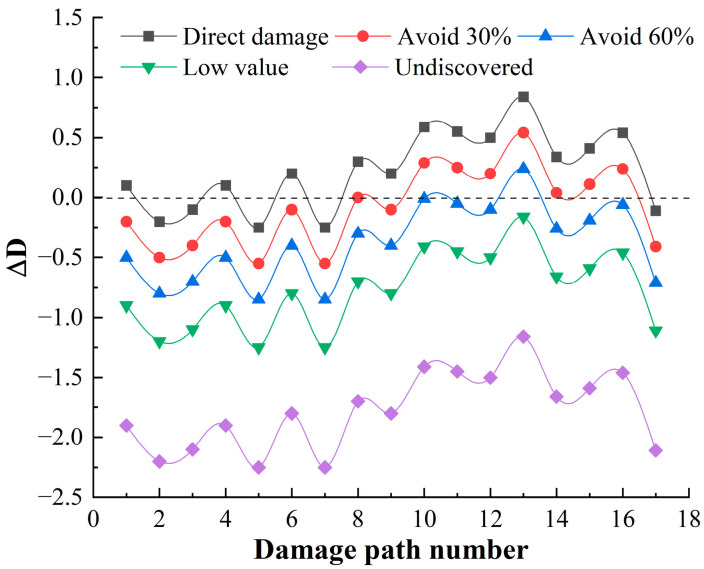
Residual damage load coefficient of mobility function.

**Figure 14:**
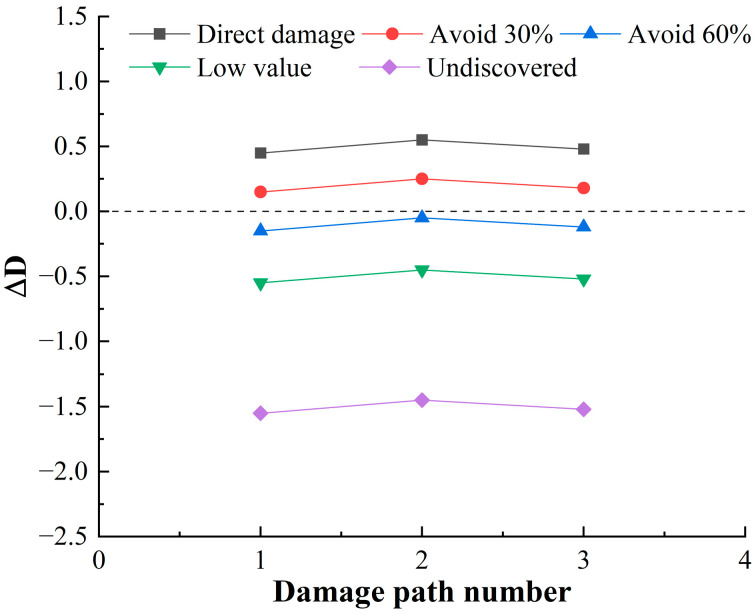
Residual damage load coefficient of communicate function.

Following the calculation process in Section 3.1, it can be calculated that there are 15 damage paths for launch function, 17 damage paths for mobility function, and 3 damage paths for communicate function, and the damage path of the mobility function accounts for the largest proportion. The residual damage load coefficient ΔD calculated on each damage path is shown in Figure 12, Figure 13 and Figure 14.

Figure 12, Figure 13 and Figure 14 shows that:

(1) When the target is discovered, and has a high value, and does not avoid direct damage, the launch function has 1 damage path with a residual damage load factor less than 0. The maneuver function has 5 damage paths with a residual damage load factor less than 0, and 0 damage paths with a residual damage load factor less than 0 for the communication function. It can be seen that the launch platform relies only on structural protection, which has little deterrent effect on damage loads, and the percentage of safe paths is 17.14%. Among these safe paths, the safe path for the launch function is 2.86%; the safe path for the mobility function is 14.28%; and the safe path for the communication function is 0, reflecting the poor protection of the launch platform.
(2) When the target is discovered and has a high value and some avoidance, that is, $1/\left[1+e^{-Q(q-q_{0})}\right]=30\%,60\%$, the value of the residual damage load coefficient begins to decrease. When the degree of avoidance is 30%, the proportion of safe paths is 42.86%; when the degree of avoidance is 60%, the proportion of safe paths is 91.43%. When the target is not discovered or the value is low, that is, ΔD<0, showing that reducing the discovery probability of the target can fundamentally block the transmission of damage load.
(3) There are 29 damage paths of ΔD≥0 when the target is discovered, and has a high value, and does not avoid direct damage, among which the damage path of the launch function accounts for the highest proportion (48.28%), which indicates that the launch function is the most likely to be damaged. In the damage path of ΔD≥0, the damage path through the cabin accounts for the highest proportion, which indicates that the damage of the launch platform is mainly from the cabin.

### 4.4. Correlation of Damage Path for HF-MCDI

**Figure 15:**
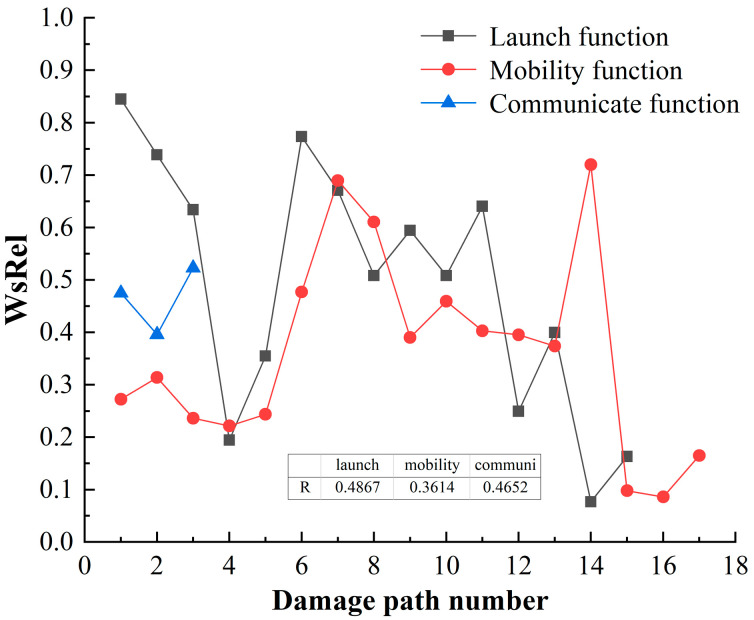
The correlation of damage path.

Following the calculation process in Section 3.2, the correlation of the damage path is shown in Figure 15.

Figure 15 shows that:

(1) On the whole, the correlation distribution between the damage and the emission function is large. The damage path of Damage load–Concealment–Value–Avoidance–Tube–Launch function has the greatest correlation, WsRel=0.8450, which indicates that damage load is most likely to destroy the function from this path.
(2) The damage path of Damage load–Concealment–Value–Avoidance–Tube–Launch function has the greatest correlation, WsRel=0.8450, which indicates that damage load is most likely to destroy the launch function from this path. The average WsRel value of launch function is 0.4901, and the correlation of the HF-MCDI of launch function is R=0.4867, which indicates that the correlation between launch function and damage load is highest, which is consistent with the damage capacity analysis results in Section 4.3.
(3) The damage path of Damage load–Concealment–Value–Avoidance–type–Mobility function has the greatest correlation in the damage path of mobility function, WsRel=0.7268, which indicates that damage load is most likely to destroy the mobility function from this path. The average WsRel value of mobility function is 0.3589, and the correlation of the HF-MCDI of mobility function is R=0.4652, which indicates that the correlation between mobility function and damage load is higher.
(4) Damage load and communication function have few damage paths, the average WsRel value of communication function is 0.4647, and the correlation of the HF-MCDI of communication function is R=0.3614.

### 4.5. Importance of Damaged Node for HF-MCDI

**Figure 16:**
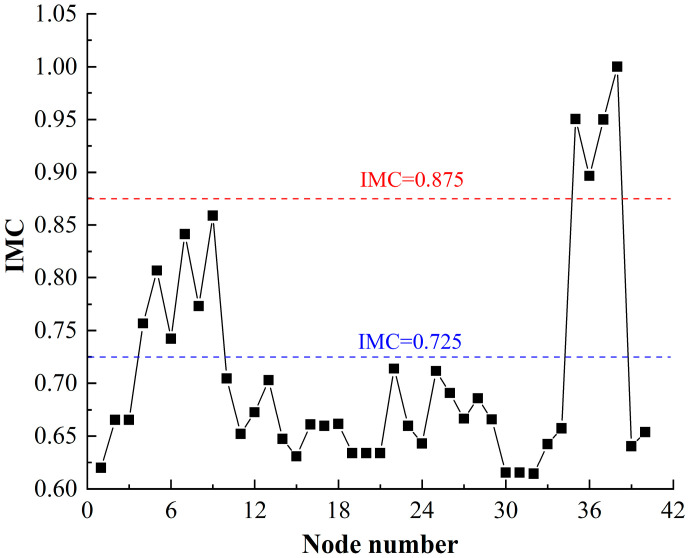
Importance of damaged node.

Following the calculation process in Section 3.3, the importance of the damaged node is shown in Figure 16.

Figure 16 shows that:

(1) The importance of damaged nodes is divided into three levels: ① There are 4 nodes with IMC values greater than 0.875, which are: Mobility function (1), Launch function (0.9681), Frame (0.9504), and Oil pipe (0.8965). This shows that these nodes are most important to the HF-MCDI of the launch platform. At the same time, it reflects that the launch platform takes the mobility function as the core function and the frame as the main structure; ② There are 6 nodes with IMC values greater than 0.725 and less than 0.875, in descending order of the largest to smallest, including the Missile, Compartment, Tube, Tyre, Cab, and Avoidance nodes, indicating that these nodes are of high importance to the HF-MCDI of the launch platform, that is, the exposed portion and avoidance have significant impact on transferring damage load; therefore, these nodes can be used in the optimization of protection; ③ For the remaining 30 nodes, the IMC value is less than 0.725 and greater than 0.6, and the importance of these nodes is small.
(2) The importance of nodes based on IMC values, which integrate the centrality of degree to measure the structure of the HF-MCDI and the centrality of betweenness to measure the transmission of the HF-MCDI, provides a more comprehensive measure of the importance of the HF-MCDI.

## 5. Conclusions

The logic of the transfer process of a damage load is analyzed, and a damage information characterization method based on heterogeneous network theory and network flow theory is established. From the obtained results, the following conclusions could be drawn.

(1) Compared with the existing methods, the proposed method can describe the damage process more completely from the viewpoint of damage transfer, and is more consistent with the actual conflict evaluation.
(2) By analyzing the basic features of the HF-MCDI, the degree distribution can indicate the structural characteristics of the damage target, the clustering coefficient can indicate the correlation characteristics of the damage information, and the node centrality can indicate the nodes with a strong influence on the damage process. The digital characterization of these fundamental features can make the study of the damage process more objective.
(3) HF-MCDI establishes the damage process in the form of a network from the initiation of the damage load to the impact on the target. The correlation of damage capacity and damage path can be used to analyze the critical links of damage information, and the importance of damage nodes can be used to analyze the critical nodes of damage information. These analyses can point out the direction for the protection of the target.

## Data Availability

Not applicable.
